# Multi‐Material 3D and 4D Printing: A Survey

**DOI:** 10.1002/advs.201902307

**Published:** 2020-04-30

**Authors:** Mohammad Rafiee, Rouhollah D. Farahani, Daniel Therriault

**Affiliations:** ^1^ Laboratory for Multiscale Mechanics Department of Mechanical Engineering Polytechnique Montreal Montreal Quebec H3T 1J4 Canada

**Keywords:** 3D printing, additive manufacturing, biomaterials, ceramics, metals, multi‐material printing, polymers

## Abstract

Recent advances in multi‐material 3D and 4D printing (time as the fourth dimension) show that the technology has the potential to extend the design space beyond complex geometries. The potential of these additive manufacturing (AM) technologies allows for functional inclusion in a low‐cost single‐step manufacturing process. Different composite materials and various AM technologies can be used and combined to create customized multi‐functional objects to suit many needs. In this work, several types of 3D and 4D printing technologies are compared and the advantages and disadvantages of each technology are discussed. The various features and applications of 3D and 4D printing technologies used in the fabrication of multi‐material objects are reviewed. Finally, new avenues for the development of multi‐material 3D and 4D printed objects are proposed, which reflect the current deficiencies and future opportunities for inclusion by AM.

## Graphical abstract

The present work reviews the state of the art in multi‐material 3D and 4D (time as the fourth dimension) printing with a special focus on polymers, ceramics and their associated composites as printing materials. This review‐type article compares the advantages and limitations of various multi‐material 3D and 4D printing technologies, represents many printed multifunctional parts for various applications ranging from aerospace to biomedical, and provides future directions the technologies discussed.

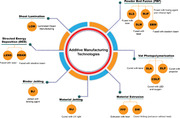

## 1 Introduction

The world's major industrial countries are promoting 3D printing or additive manufacturing (AM) as a technology foundation of future manufacturing. Due to special characteristics of AM such as facile and customizable manufacturing, this method is being broadly used in many areas such as electronics, aerospace, robotics, and textile.^[^ ^1^ ^]^ With the emerging of smart materials, attempts to combine them with AM led to 3D parts that are activated by external stimuli and/or environment over time (i.e., 4D (4‐dimensional) printing).^[^ ^2^ ^]^ Current initiatives in the development of AM tools involve development of multi‐material 3D and 4D printing. Using multi‐material 3D and 4D printing, it is feasible to ameliorate the quality of parts by altering composition or type of materials within the layers; that is not easy to obtain by conventional manufacturing methods. A wide range of materials such as polymers, metals, ceramics, and biomaterials has been used in various AM methods to obtain multi‐material products. Therefore, a thorough understanding of multi‐material 3D and 4D printing is required.

The number of research pertaining the additive manufacturing of multi‐material parts has steadily increased since 2010. Some surveys of multi‐material AM already exist in the literature. For example, review articles^[^ ^3^, ^4^, ^5^ ^]^ covered some of the research done on the multi‐material printing prior to 2017. In response to the growing interest in this area, the present article aims to provide a broader and updated review on 3D and 4D printing of multi‐material parts, by providing a comprehensive list of multi‐material additive manufacturing methods published in the literature.

Here, we review the technologies and applications of multi‐material 3D and 4D printing. We first consider the main technologies for printing multi‐material objects. Next, we describe the multi‐material 3D and 4D printing for different types of materials: polymers, metals, ceramics, and biomaterials. Finally, we discuss the limitations of current technologies and the challenges for future research. To limit the scope of our survey, the emphasis has been on the additive manufacturing of parts made of discrete multiple materials. Publications directly related to other aspects of multi‐material additive manufacturing, such as the raw materials are premixed or composited before the 3D printing, or porous materials suitable for secondary material infiltration have been excluded from this survey.

In the current survey, some of the publications may have multiple citations. For instance, a paper in which both polymer and biomaterial study are presented will be cited in the “Multi‐material additive manufacturing of polymers” as well as the “Multi‐material additive manufacturing of biomaterials” sections.

## 2 Multi‐Material Additive Manufacturing Technologies

**Figure 1:**
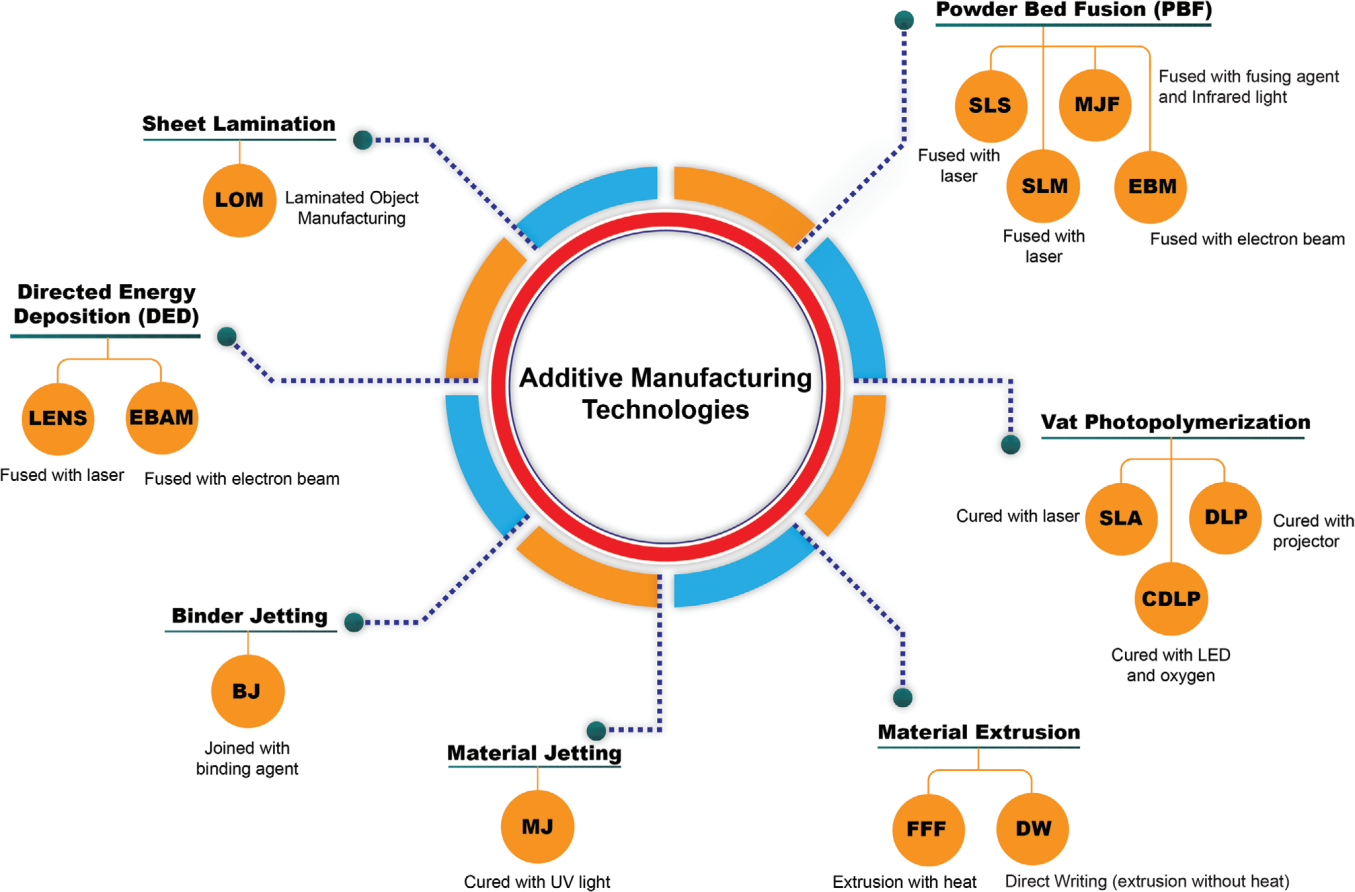
Classification of additive manufacturing technologies; the seven categories: material extrusion, vat photopolymerization, binder jetting, material jetting, sheet lamination, directed energy deposition, and powder bed fusion.

Multi‐material additive manufacturing systems may be classified based on the technology, feed stock, source of energy, build volume, etc. Based on the ISO/ASTM 529000:2015 standard, AM methods can be classified into seven different categories and examples of AM processes are depicted in **Figure** **1**.

Multi‐material additive manufacturing technology can reduce production time with no extra cost for manufacturing parts with complex morphology. As shown in Figure 1, seven AM technologies are currently available commercially, with each having its own advantages and limitations. An overview of some of these technologies and a summary of their main advantages and disadvantages are provided in **Table** **1**. Although there is a variety of commercially available 3D printers, only a limited number enables the production of 3D parts with multiple materials. **Tables** **2** and **3** list some of the commercially available multi‐material 3D printers and their specifications for polymers and biomaterials, respectively.

**Table 1:** Additive manufacturing technologies

Technology	Method	Process description	Advantages/disadvantages	Application areas
Vat photopolymerization	Stereolithography (SL) Synonyms: SLA	SL makes use of a photopolymer liquid as the source material in a vat. This liquid plastic is transformed into a 3D object layer‐by‐layer by lowering the build platform into the vat and curing using a UV laser.	⊕ Can build large parts with very good accuracy and surface finish ⊖ Works with photopolymers which are not stable over time and do not have well defined mechanical properties.	Prototypes, casting patterns, jewelry, dental, and medical applications
	Digital light processing (DLP)	DLP technology is very similar to SL but uses a different light source and makes use of a liquid crystal display panel.	⊕ Higher print speed compared with SLA ⊕ Excellent accuracy of laying ⊕ Low cost printers ⊖ Insecurity of the consumable material ⊖ High cost of materials	Prototypes, casting patterns, jewelry, dental, and medical applications
	Continuous direct light processing (CDLP)	CDLP works similar to DLP except it relies on the continuous motion of the printing bed in the *z*‐direction (upward). Faster build times are possible as the printer does not have to stop and separate the object from the printing bed after each layer is printed.	⊕ High print speed ⊕ Excellent accuracy of laying ⊕ Low cost printers ⊖ Insecurity of the consumable material ⊖ High cost of materials	Prototypes, casting patterns, jewelry, dental, and medical applications
Material extrusion	Fused deposition modeling (FDM) Synonyms: Fused filament fabrication, FFF Fused layer modeling/manufacturing, FLM	A plastic filament is melted and extruded through a nozzle. Objects are built layer‐by‐layer.	⊕ Can build fully functional parts in standard plastics ⊖ Printed parts have an anisotropy in the *z*‐direction (vertical direction) and a step‐structure on the surface	Prototypes, support parts (jigs, fixtures), small series parts
	Direct ink writing (DIW) Synonyms: Robocasting (RC), direct‐write assembly (DWA), or microrobotic deposition (μRD), bioplotting, low‐temperature deposition manufacturing (LDM), freeform 3D printing, extrusion freeform fabrication (EFF)	Material in a semi‐liquid or paste form can be extruded through a nozzle and used to print the cross sections of a sliced 3D model.	⊕ Highest resolution for an extrusion system ⊕ Ideal for research environments and medical (bone) applications ⊖ Limited part geometry ⊖ High cost of system ⊖ Small build volume	Solid monolithic parts, scaffolds, biologically compatible tissue implants, tailored composite materials, ceramics
Binder jetting (BJ)	3D printing, BJ	Inkjet printing heads jet a liquid‐like bonding agent onto surface of powder. By bonding the particles together, the object is built up layer‐by‐layer.	⊕ A rather fast and cheap technology ⊕ Wide range of material types ⊕ Parts in full color are possible ⊖ Parts coming directly from the machine have limited mechanical properties	Prototypes, casting patterns, molds and cores
Material jetting (MJ)	Multijet modeling, drop on demand, DOD, thermojet, inkjet printing	Inkjet printing head jets molten wax onto a printing bed. Once the material is cooled and solidified, it allows to fabricate layers on top of each other.	⊕ Can achieve very good accuracy and surface finishes ⊖ Only works with wax‐like materials	Prototypes, casting patterns
	Polyjet modeling, multijet modeling, polyjetting, multijetting, jetted photopolymer	Similar to multijet except printing head jets liquid photopolymers onto a printing bed. The material is immediately cured by UV light and solidified which allows to build layers on top of each other.	⊕ Different materials can be jetted together to achieve multi‐material and multi‐color objects ⊖ Works with UV‐active photopolymers which are not durable over time	Prototypes, casting patterns, tools for injection molding
Powder bed fusion (PBF)	Laser sintering (LS) Synonyms: Selective laser sintering, SLS	SLS has some similarities with SL. A thin layer of plastic powder is selectively melted by a laser. The parts are built up layer‐by‐layer in the powder bed.	⊕ Can manufacture parts in standard plastics with good mechanical properties ⊕ A constantly growing set of materials available ⊖ Parts do not have exactly the same properties as their injection molded counterparts	Prototypes, support parts, small series parts
	Selective laser melting, SLM; direct metal laser sintering, DMLS; laser cusing	The LS process is very similar to the LM process. A thin layer of metal powder is selectively melted by a laser. The parts are built up layer by layer in the powder bed.	⊕ Can manufacture parts in standard metals with high density, which can be further processed as any welding part ⊖ Is rather slow and expensive ⊖ Surface finishes are limited	Prototypes, support parts (jigs, fixtures, etc.), small series parts, tools
	Electron beam melting (EBM)	A thin layer of metal powder is selectively melted by an electron beam. The parts are built up layer by layer the in the powder bed.	⊕ Parts can be manufactured in some standard metals with high density by electron beam melting ⊖ The availability of materials is limited ⊖ The process is rather slow and expensive	Prototypes, small series parts, support parts
	Multijet fusion (MJF)	MJF is basically a combination of the SLS and MJ technologies. A carriage with inkjet nozzles deposits fusing agent on a thin layer of plastic powder in which it selectively melted with a high‐power IR energy source.	⊕ High production speed ⊖ The availability of materials is very limited	Prototypes, production parts, housings
Directed energy deposition (DED)	Laser engineered net shaping (LENS)	Uses a high power laser to melt metal powder that is deposited onto the table. Metal is sprayed onto the focal point on the laser where the metal becomes fused together. An inert gas is used to shield the metal from atmospheric gases. It uses a layered approach to manufacture the components.	⊕ Can be used to repair parts as well as fabricate new ones ⊕ Has a very good granular structure ⊕ Powder forming methods have only few material limitations ⊕ The properties of the material are similar or better than the properties of the natural materials ⊖ Some post‐processing involved ⊖ The part must be cut from the build substrate ⊖ Has a rough surface finish, ⊖ May require machining or polishing ⊖ Low dimensional accuracy	Fabrication and repair of injection molding tools, fabrication of large titanium and other exotic metal parts for aerospace applications
	Electron beam additive manufacture (EBAM)	Uses an electron beam as the heat source to weld and create metal parts using wire or metal powder. The method is similar to LENS, however, electron beams are more efficient than lasers.	⊕ A wider selection and greater availability of wire products versus powder ⊕ Wire feedstock is cheaper than powder ones ⊕ Less safety and procurement issues compared with LENS ⊕ Significantly less energy consumption compared with powder‐feed method ⊖ Limited to single material printing	Fabrication and repair of injection molding tools, fabrication of large titanium and other exotic metal parts for aerospace applications
Sheet lamination	Laminated object manufacturing (LOM)	Layers of paper, plastic, or metal laminates are coated with adhesive and welded together using heat and pressure and then cut to shape with a computer controlled laser or knife.	⊕ Ability to produce larger‐scaled models ⊕ Uses very inexpensive paper ⊕ Fast and accurate ⊕ Good handling strength ⊖ Need for decubing, which requires a lot of labor, can be a fire hazard, and finish, accuracy and stability of paper objects ⊖ Not as good as materials used with other rapid prototyping methods	Prototypes, large parts

**Table 2:** Multi‐material polymer and polymer composite 3D printers

Technology	3D printer commercial name/Manufacturer (Country)	Build volume [mm^3^]	Nozzle type	Layer resolution [mm]	Stock materials	Open source
Material extrusion (FDM)	Duplicator 5/Geeetech (China)	230 × 150 × 150	Dual	0.1–0.3	Filament: ABS/PLA/flexible PLA/wood /nylon	No
	Creater Pro/FlashForge (China)	227 × 148 × 150	Dual	0.1 ≈ 0.5	ABS/PLA	No
	CraftBot3/CraftBot (Hungary)	270 × 250 × 250	Dual (separate)	0.1 ≈ 0.3	N/A	No
	BCN3D SIGMA R19/BCN3D Technologies (Spain)	210 × 297 × 210	Dual (separate)	0.05–0.5	PLA/ABS/nylon/PET‐G/TPU/PVA/composites/others	Yes
	Zortrax Inventure/Zortrax (Poland)	135 × 135 × 130	Dual	0.09–0.29	Model materials (Z‐PETG, Z‐PLA, Z‐SEMIFLEX, Z‐ULTRAT Plus) and support materials (Z‐SUPPORT, Z‐SUPPORT Plus)	No
	Makergear M3‐ID/Makergear	Head 1: 203 × 232 × 203, Head 2: 180 × 232 × 203	Dual (separate)	0.02–0.35	ABS, ASA, HIPS, Nylon, PET‐G, PET‐T, PLA, polycarbonate, polypropylene, PVA, TPE, TPU, metal composites, wood composites, carbon fiber composites	No
	Ultimaker 3/Ultimaker	197 × 215 × 200	Dual	0.02–0.6	PLA, tough PLA, ABS, nylon, CPE, CPE+, PC, PP, TPU 95A, PVA	Yes
	3DWOX 2X/Sindoh	228 × 200 × 300	Dual (separate)	0.05–0.4	PLA, ABS, flexible, PVA	No
	Raise3D Pro2/Raise3D	280 × 305 × 300	Dual	N/A	PLA/ABS/HIPS/PC/TPU/TPE/NYLON/PETG/ASA PP/glass fiber enforced/carbon fiber enforced Metal particles filled/wood fille	No
	LulzBot TAZ Workhorse/LULZBOT (USA)	280 × 280 × 285	Dual	0.05–0.4	PLA, ABS, nylon, polycarbonate, carbon fiber reinforced blends, TPU 85A and 95A (flexible), PETG, PETT, copolyester, PVB (polycast), PVA, HIPS, and many more 3rd party filaments	Yes
	ZMorph VX/ZMorph (Poland)	250 × 235 × 165	Dual	0.05 ≈ 0.4	ABS, PLA, PVA, PET, ASA, nylon, HIPS, thermochrome, TPU, flex materials	No
	CEL RoboxPRO/CEL (UK)	210 × 300 × 400	Dual	0.05 ≈ 0.5	ABS, PETG, PC, nylon, PVOH	No
	Ultimaker S5/Ultimaker	330 × 240 × 300	Dual	0.02–0.6	PLA, tough PLA, ABS, nylon, CPE, CPE+, PC, PP, TPU 95A, PVA	Yes
Material jetting	ProJet® MJP 5600/3D systems (USA)	518 × 381 × 300	N/A	.013–016	Flexible and rigid photopolymers within the VisiJet family of materials	No
	Objet260 Connex3/Stratasys (USA)	255 × 252 × 200	N/A	0.016	Variety of materials such as Vero family	No
	J735/Stratasys (USA)	350 × 350 × 200	N/A	0.014	Variety of materials such as Vero family	No
	J750/ Stratasys (USA)	490 × 390 × 200	N/A	0.014	Variety of materials such as Vero family	No
	OBJET1000 PLUS/Stratasys (USA)	1000 × 800 × 500	N/A	0.016	Variety of materials such as Vero family	No
	Objet Connex350/ Stratasys (USA)	342 × 342 × 200	N/A	0.016	Variety of materials such as Vero family	No
	Objet Connex500/Stratasys (USA)	490 × 390 × 200	N/A	0.016	Variety of materials such as Vero family	No
	F900/Stratasys (USA)	914.4 × 609.6 × 914.4	N/A	0.127–0.508	Variety of materials such as Vero family	No
	Multi‐Fab/Computational Fabrication Group, Massachusetts Institute of Technology (USA)	N/A	N/A	N/A	Variety of materials	Yes
FDM and MJ (curing by UV)	3Dn DDM/nScrypt (USA)	300 × 300 × 150	Up to 5	0.0005	Variety of third party materials for both UV assisted and FDM processes	Yes
FDM and continuous filament fabrication (CFF)	Onyx Pro (Desktop)/Markforged (USA)	320 × 132 × 154	1	0.1	Onyx fiber materials: continuous fiberglass	No
	Mark Two (Desktop)/Markforged (USA)	320 × 132 × 154	1	0.1	Onyx fiber materials: carbon fiber, fiberglass Kevlar, HSHT fiberglass (high‐strength high‐temperature fiber‐glass)	No
	MARKFORGED X5 (Desktop) / Markforged (USA)	330 × 270 × 200	1	0.1	Onyx fiber materials: continuous fiberglass	No
	MARKFORGED X7 (Desktop)/Markforged (USA)	320 × 132 × 154	1	0.1	Onyx fiber materials: carbon fiber, fiberglass Kevlar, HSHT fiberglass (high‐strength high‐temperature fiber‐glass)	No

**Table 3:** Multi‐material biomaterial 3D printers

Technology	3D printer	Build volume [mm^3^]	Printing head	Layer resolution [mm]	Stock materials	Open source
Material extrusion (DIW)	3D‐Bioplotter Starter series/ EnvisionTEC (Germany)	150 × 150 × 80	2	0.1	Any liquid, melt, paste, or gel can be used to be dispensed through a needle tip	Yes
	3D‐Bioplotter Developer series/EnvisionTEC (Germany)	150 × 150 × 140	Up to 3	0.1	Any liquid, melt, paste, or gel can be used to be dispensed through a needle tip	Yes
	3D‐Bioplotter Manufacturer series/EnvisionTEC (Germany)	150 × 150 × 140	Up to 5	0.1	Any liquid, melt, paste, or gel can be used to be dispensed through a needle tip	Yes
	BioFactory/RegenHU (Switzerland)	60 × 55 × 55	Up to 8	N/A	Any liquid, melt, paste, or gel can be used to be dispensed through a needle tip	Yes
	3Ddiscovery (Bench‐top)/RegenHU (Switzerland)	130 × 90 × 60	Up to 7	N/A	Any liquid, melt, paste, or gel can be used to be dispensed through a needle tip	Yes
	BioScaffolder 3.2 and 4.2/GESIM (Germany)	N/A	3	N/A	Any liquid, melt, paste, or gel can be used to be dispensed through a needle tip	Yes

## 3 Multi‐Material Additive Manufacturing of Polymers

There have been significant efforts in the scientific community to fabricate multi‐material polymer composites. In this section, we review the related works on polymers and their composites.

### 3.1 Vat Photopolymerization

A vat of liquid photopolymer (resin) is used by vat photopolymerization, and the model is printed layer by layer using some types of light sources. Stereolithography (SL), digital light processing (DLP), and digital light synthesis (DLS) are the three main vat polymerization techniques. The vat photopolymerization process is not generally a candidate for multi‐material 3D printing. It constructs parts from a vat of photopolymers, and thus using multiple materials in vat photopolymerization provides difficulties with controlling contamination between each vat. However, due to its advantages such as surface finish, accuracy of dimensions, and the options for a variety of materials, vat photopolymerization has been adapted to support multi‐material printing.^[^ ^6^, ^7^, ^8^, ^9^, ^10^, ^11^, ^12^, ^13^, ^14^, ^15^, ^16^ ^]^ This is achieved by using multiple vat systems with different UV‐curable polymers. These systems can provide high printing resolution, but changing materials during printing significantly slows down the printing process.^[^ ^17^ ^]^

3D printing of short and continuous fiber‐reinforced polymer composites using SL was studied by Sano et al.^[^ ^14^ ^]^. Glass powder and fiberglass fabric were used as the short and continuous fiber reinforcement of light‐cured resin materials. The tensile strength and Young's modulus were 7.2 and 11.5 times higher than those of the pure resin specimens, respectively.

**Figure 2:**
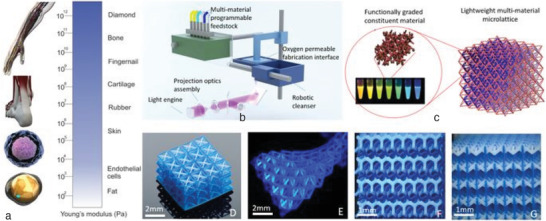
3D printing of multi‐material microscale lattice with dissimilar materials using digital light projection micro‐stereolithography approach (PµSL): a) 3D multi‐material microscale lattice, b) PµSL setup, c) bimaterial microlattice, d–g) isotropic microscale lattice comprised of different polymers. Reproduced with permission.^[^ ^16^ ^]^ Copyright 2018, Springer Nature.

Digital light projection micro‐stereolithography (PµSL) is an additive micro manufacturing method capable of manufacturing arbitrary 3D micro‐scale structures. Using PµSL, Chen and Zheng^[^ ^16^ ^]^ fabricated multi‐material metamaterials with big and tailorable negative Poisson's ratios (**Figure** **2**). Their multi‐modulus metamaterials were comprised of encoded elasticity ranging from soft to rigid. The authors found that, in contrast to ordinary architected materials whose negative Poisson's ratio is governed by their geometry, these metamaterials are capable of exhibiting Poisson's ratios from large negative to zero, independent of their 3D micromechanical structure.

### 3.2 Material Extrusion

The core principle of material extrusion‐based technologies is that any material that is in a paste or semi‐liquid form can be extruded through a nozzle and used to build layer‐by‐layer a sliced 3D model. Depending on the temperature required or suitable for the extrusion, it can be classified into two main sub‐groups: fused filament fabrication (FFF) or fused deposition modeling (FDM) for extrusion of melted thermoplastic polymers and direct ink writing (DIW) for extrusion without melting. Different terminologies associated with these two categories are provided in Table 1. The material extrusion technology can be easily extended to multi‐material 3D printing through the use of multiple nozzles.

#### 3.2.1 Fused Filament Fabrication

**Figure 3:**
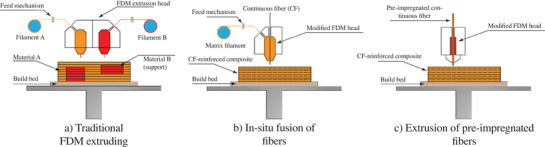
Extrusion based multi‐material additive manufacturing: a) traditional FDM; b) in situ fusion of fibers with molten thermoplastic in the nozzle; c) extrusion of pre‐impregnated fibers.

The FFF uses filaments made of thermoplastic polymers which are melted and extruded through a nozzle on the desired substrate in a layer‐by‐layer manner. Dual or multi‐extruder printing heads are often used in material extrusion systems to print multi‐material parts at once. For example, it is a common method to use one extruder to print dissolvable supports that can be easily removed from the main printed structure, or use them to print in two colors, or two materials that will be present in the end print (see **Figure** **3**a). Many multi‐material FFF printers are listed in Table 2. However, dual and multi‐extruder printers typically come with a few limitations: the presence of the additional extruder (second one or more) will reduce the printing area that would be available for printing with a single extruder; the chances of oozing and stringing become higher; and finally layer‐shifting defects may be observed if one of the extruders causes material deposited by the other to warp.

Recently, some research groups have improved the mechanical performance of 3D printed polymers by reinforcing them with continuous fibers. Printing using continuous fibers have also been tried using customized FDM printers using polylactic acid (PLA),^[^ ^18^, ^19^, ^20^, ^21^ ^]^ Acrylo Butadien Styrene (ABS),^[^ ^20^, ^21^, ^22^, ^23^ ^]^ nylon,^[^ ^24^, ^25^, ^26^, ^27^, ^28^, ^29^, ^30^ ^]^ and epoxy resin^[^ ^31^ ^]^ as the matrix, while carbon,^[^ ^18^, ^19^, ^20^, ^21^, ^23^, ^24^, ^26^, ^27^, ^28^, ^29^, ^30^, ^31^, ^32^ ^]^ glass,^[^ ^27^, ^33^ ^]^ and Kevlar^[^ ^25^, ^27^ ^]^ fibers have been used as the reinforcements. Two main configurations for printing continuous fiber‐reinforced composites have been developed as i) in‐situ fusion of fibers with thermoplastic in the nozzle ^[^ ^18^, ^20^, ^22^, ^23^, ^24^, ^26^, ^27^, ^28^, ^30^, ^32^ ^]^ and ii) extrusion of pre‐impregnated fibers.^[^ ^21^, ^29^ ^]^ The former approach (See Figure 3b) can be performed by modifying the printing head to receive the continuous fiber and thermoplastic filament, simultaneously. One of the main challenges of the first approach is to have a proper bonding between the reinforcement and the matrix. This is mainly because the printing head cannot generate enough pressure to push the melting resin onto the reinforcing fiber; besides, the short time of impregnation of continuous fibers is another reason. The second approach (see Figure 3c) is much more complicated compared with the first one, and good impregnation of long fibers is not certain.

The method of in situ fusion of fibers with molten thermoplastics was often used by researchers for additive manufacturing of continuous fiber composites. This approach was used by the Markforged company (USA) as the manufacturer of the most common commercially available multi‐material continuous fiber composite printers.^[^ ^24^, ^25^, ^26^, ^27^, ^28^ ^]^ The Markforged line of 3D printers is limited to their own 3D printing Eiger software, each printer only uses one type of specialized and expensive filament, the carbon fiber inlay method and a few other parts of the printer are locked‐down by some patents. Several authors used Markforged 3D printers (Mark One,^[^ ^24^, ^25^, ^26^, ^27^ ^]^ Mark Two,^[^ ^28^ ^]^ and Mark X^[^ ^30^ ^]^) for the fabrication of continuous fiber‐reinforced multi‐material composite. For instance, Peng et al.^[^ ^30^ ^]^ studied the effect of synergistic reinforcement on the mechanical properties of additively manufactured polyamide‐based composites filled with continuous and short carbon fibers. Morphological, thermal, and mechanical testing for the printing tows were first characterized to obtain the properties of the printing materials. The mechanical properties of laminated composites showed to be higher with increasing continuous carbon fiber content.

Aside from Markforged printers, there have been other customized 3D printers that used the approach of in situ fusion of fibers with molten thermoplastics for additive manufacturing of continuous fiber composites.^[^ ^18^, ^20^, ^22^, ^23^, ^32^ ^]^ For instance, Nakagawa et al.^[^ ^32^ ^]^ improved the strength of printed thermoplastic parts by sandwiching continuous carbon fibers between upper and lower ABS layers. An FDM‐based 3D printer for manufacturing of continuous fiber‐reinforced thermoplastic composites was developed by Yang et al.^[^ ^23^ ^]^. The authors also developed an extrusion head for the continuous fiber hot‐dipping purpose. Their work showed that the bending and tensile strength of these 10 wt% continuous carbon fiber/ABS specimens were improved to 127 and 147 MPa, respectively. These values were far greater than the ABS parts and close to the continuous carbon fiber/ABS composites manufactured by injection molding process with the same fiber content.

The extrusion of pre‐impregnated fibers was also used by some researchers due its advantages such as achieving better bonding between the matrix and the continuous fibers. Fabrication and 3D printing of continuous carbon fiber prepreg filaments were performed by Hu et al.^[^ ^21^ ^]^. The flexural properties of parts printed with the filament were studied. It was found that layer thickness has a significant influence on the final strength and modulus, while the printing temperature and speed had minor influences. By using FDM approach, combined with a continuous toolpath (G‐code), Dickson et al.^[^ ^29^ ^]^ produced woven continuous carbon fiber composites. Studies on open hole tensile coupons were conducted in which 6 mm holes were routed into the fiber‐reinforced composite structure and the resulting mechanical performance of the parts were compared with specimens which had been die‐punched as well as an un‐notched control group. The latter showed a strength equivalent to 49% that of unnotched specimen.

**Figure 4:**
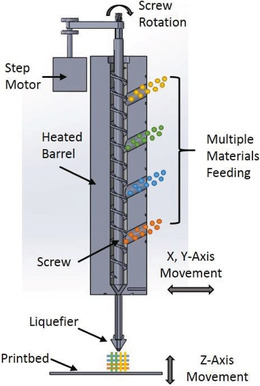
Schematic of a single screw extrusion‐based AM system. Reproduced with permission.^[^ ^34^ ^]^ Copyright 2016, AIP Publishing.

FDM‐based multi‐material additive manufacturing through a single extruder was also reported in the literature.^[^ ^34^ ^]^ In this direct feed FDM technology (**Figure** **4**), multiple materials in any available form can be co‐fed into a single‐screw extruder and subsequently deposited onto the print bed. This technology potentially has the capability to print a structure with controllable and variable compositions.

**Figure 5:**
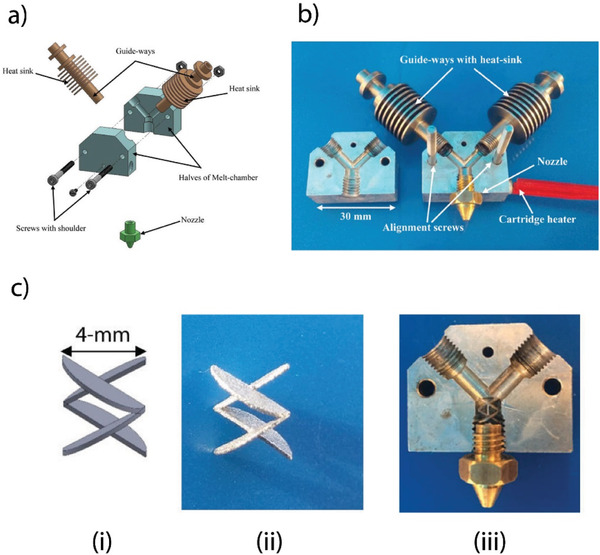
Custom bi‐extruder for FDM multi‐material additive manufacturing of FGM objects: a) an exploded view of the designed bi‐extruder, b) assembly of the manufactured bi‐extruder, c‐i) the 3D model of the passive inter‐mixer, c‐ii) image of the inter‐mixer fabricated by DMLS, c‐iii) an image showing the inter‐mixer inserted into the bi‐extruder channel. Adapted with permission.^[^ ^35^ ^]^ Copyright 2018, Emerald Publishing Limited.

Khondoker et al.^[^ ^35^ ^]^ proposed a customized bi‐extruder for FDM multi‐material additive manufacturing of functionally graded materials (FGMs) made of immiscible thermoplastics (see **Figure** **5**). The proposed bi‐extruder can print two thermoplastic polymers through a single nozzle with a static inter‐mixer to enhance the adhesion between feeding materials. The dual‐extruder was characterized by printing parts using PLA, ABS, and high impact polystyrene (PS). It was also observed that the mechanically interlocked extrudates substantially reduce adhesion failures within and between filaments.

The capability of printing complicated patterns of multiple materials by combining multiple printing heads has been useful for antenna engineers. This approach provided them with a powerful tool to rapidly prototype new antenna concepts and the ability to explore ideas not realizable using standard fabrication processes.^[^ ^36^, ^37^, ^38^ ^]^ An FDM‐based multi‐material additive manufacturing printer (3Dn‐300) sold by nScrypt Inc (see Table 2) was used by some authors to fabricate antennas.^[^ ^36^, ^37^, ^38^ ^]^ For instance, Pa et al.^[^ ^36^ ^]^ fabricated a low‐profile antenna that includes an integrated artificial magnetic conducting (AMC) ground plane. This system integrates dual deposition heads in which one print head dispenses Polycarbonate (PC) using a filament extrusion process to print all the dielectric components and the second head prints silver conductive elements using a micro‐dispensing technology.

#### 3.2.2 Direct Ink Writing

**Figure 6:**
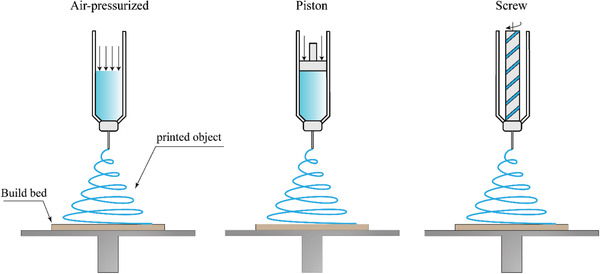
DIW printers use pressurized air, piston, or screw for extrusion of materials.^[^ ^115^ ^]^

In multi‐material DIW, the paste‐like materials are extruded which do not necessarily need to be polymers or even to be heated. The material filament is deposited using dispensers (usually pneumatic dispensers) that are mounted onto a motion‐controlled positioning stage or a dispensing robot (**Figure** **6**). The printing materials, such as epoxy resins, however, requires certain viscoelastic and rheological characteristics to be smoothly extruded from the printing head. The majority of ink solutions made using such materials show a shear‐thinning rheological behavior characterized by decreasing viscosity with increasing shear rate.

**Figure 7:**
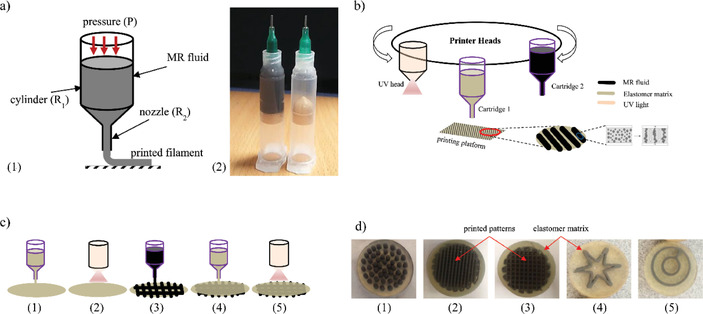
a) 1) Schematic of the MR fluid printing system including a piston‐cylinder unit and a printing nozzle, 2) printing cartridges with MR fluid (black) and elastomer matrix (clear); b) schematic for printing of hybrid MR elastomer via DIW; c) the steps involved in printing of hybrid MR elastomer: 1) elastomer matrix deposition to form a bottom layer, 2) bottom layer curing with UV light, 3) printing of MR fluid, 4) elastomer matrix deposition to cover MR fluid patterns, 5) curing with UV light; and d) 3D printed hybrid MR elastomers: 1) dot pattern, 2) line pattern, 3) line pattern with mesh, 4) asterisk shaped pattern, 5) circular pattern; Adapted with permission.^[^ ^43^ ^]^ Copyright 2018, Elsevier.

Oxman et al.^[^ ^39^ ^]^ proposed a DIW‐based AM platform for the fabrication of FGMs. In their study, a printing head consisting of a nozzle with a mixing unit was installed to the *z*‐direction of a gantry robotic machine. The authors printed silicone in two different colors to demonstrate the performance of their 3D printer. Rocha et al.^[^ ^40^ ^]^ fabricated graphene‐based electrodes for electrochemical energy storage using inks with thermoresponsive properties. Reduced chemically modified graphene (rCMG) was incorporated in their polymer composites (e.g., Pluronic F127; BASF) for enhancement of thermo‐electrical properties. The electrochemical performance of their rCMG‐based electrode demonstrated the potential of multi‐material printing in energy applications. Kikkinis et al.^[^ ^41^ ^]^ used a DIW‐based 3D printer (3D discovery from RegenHU, see Table 3) for multi‐material additive manufacturing of heterogeneous composites under an external magnetic field. Bastola et al.^[^ ^42^, ^43^ ^]^ fabricated multi‐material hybrid magnetorheological elastomers using a BioFactory 3D printer made by RegenHU (Switzerland). In their work, a controlled volume of a magnetorheological (MR) fluid was encapsulated layer by layer into an elastomer (silicone) matrix as shown in **Figure** **7**.

3D printing of fiber‐reinforced thermosetting composites were also reported in the literature. A custom‐made 3D printing platform was used in the work of Hao et al.^[^ ^31^ ^]^ to print continuous fiber‐reinforced epoxy composites. The mechanical properties of the composite lamina were characterized in their study. Their results indicated that the mechanical properties of the fabricated epoxy composite were better than that of similar PLA and short carbon fiber reinforced composite ones.

Li et al.^[^ ^44^ ^]^ combined DIW and microfluidics to manufacture a multi‐material 3D printing system for printing textured composites with liquid inclusions of programmable compositions and distributions. The printing system used was based on commercial LulzBot (Aleph Objects) printer with its original printhead replaced by Objet350 Connex3 printer. Microfluidic chips and the nozzle were integrated to the printhead. The proposed multi‐material microfluidic 3D printing framework could be used to fabricate soft robotic devices.

**Figure 8:**
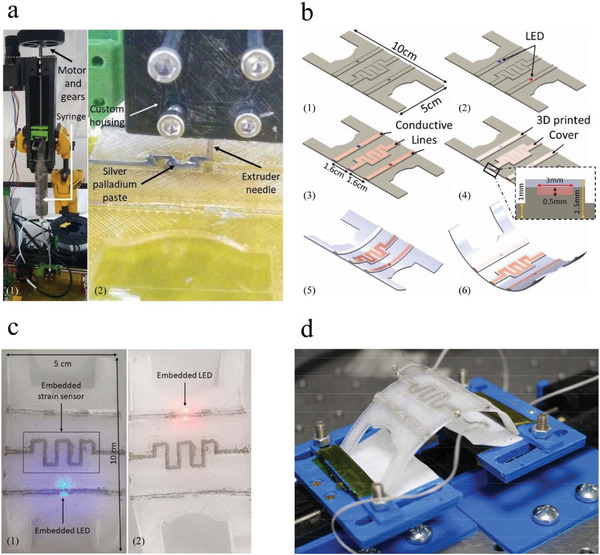
Smart flexible sensing circuit using a modified 3D printer: a) 1) extrusion system for conductive paste, 2) syringe and custom housing for the extrusion mechanism; b) CAD design of the printed structure: 1) the bottom layer with empty cavities, 2) placement of the colored LEDs, 3) silver‐palladium paste printed, 4) structure with the top plastic layer printed embedding the sensor and electronics, 5) *y*‐axis bending, 6) *x*‐axis bending; c) 1) fabricated multi‐material 3D printed smart sensing structures with the fully embedded blue LED, 2) testing of the fully embedded red LED; and d) Bending test set up to evaluate the embedded printed strain sensor. Adapted with permission.^[^ ^45^ ^]^ Copyright 2018, IEEE.

Nassar et al.^[^ ^45^ ^]^ used a fully open‐source DIW 3D printer to fabricate flexible smart sensors as shown in **Figure** **8**. The authors modified a RepRap Pro Ormerod 2 desktop 3D printer to include a second printing head for the extrusion of pastes and inks. A silver palladium paste mixed with ethanol was used as the conductive material and Glassbend Flexi was used as the flexible substrate material. With a single‐step procedure for simultaneous printing of structural and functional materials, the authors demonstrated the feasibility of fabricating complex packages with embedded sensing and electronic components.

**Figure 9:**
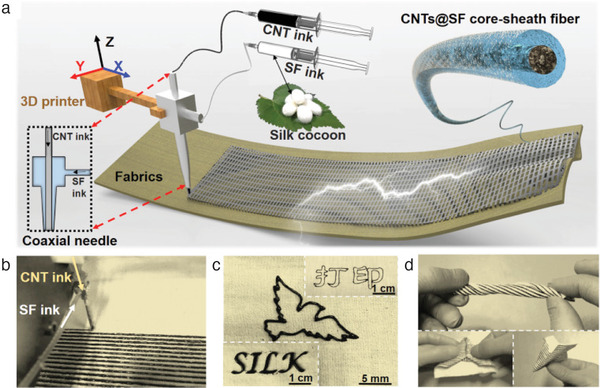
DIW of core‐shell patterns on fabrics: a) a schematic depicting the coaxial 3D printing; b) picture of the 3D printing process; c) some printed patterns; d) a picture showing the flexibility of the printed textile. Reproduced with permission.^[^ ^46^ ^]^ Copyright 2019, Elsevier.

**Figure 10:**
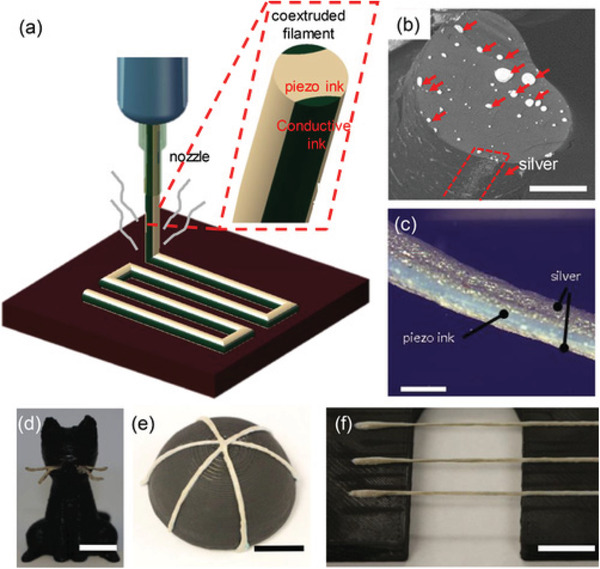
a) Schematic of the coaxial printing process, inset: cross section of the coextruded filament illustrating the piezoelectric in the core and the conductive inks as the shell; b) SEM image of cross section of the coextruded filament (scale bar  =  1 mm); c) picture of the coextruded piezoelectric thread (scale bar  =  500 µm); d) freestanding whiskers printed on a FDM printed cat (scale bar  =  10 mm); e) conformal sensors printed on a hemisphere (scale bar  =  5 mm); and f) spanning filaments (scale bar  =  10 mm). Reproduced with permission.^[^ ^47^ ^]^ Copyright 2018, John Wiley and Sons.

Coextrusion of inks has led to 3D printing of wearable textile and sensors. Zhang et al.^[^ ^46^ ^]^ developed a single‐step printing of fiber‐reinforced smart patterns for electronic textile (E‐textile) using a Anycubic I3 MEGA 3D printer equipped with a coaxial spinneret as shown in **Figure** **9**. The authors used silk fibroin and CNT ink as the shell and core layer, respectively. In another work, Bodkhe et al.^[^ ^47^ ^]^ used DIW to 3D print piezoelectric sensors with their coextruded silver electrodes in a single step (see **Figure** **10**). In their work, an I&J 2200–4 (I&J Fisnar) robotic 3D printer was used to coextrude PVDF/BaTiO_3_ nanocomposites with a commercially available silver ink to fabricate piezoelectric sensors (Figure 10b). Their printed piezoelectric sensors successfully worked and the produced voltage was linearly proportional to the applied strain.

##### Multi‐Material FGMs in Material Extrusion

**Figure 11:**
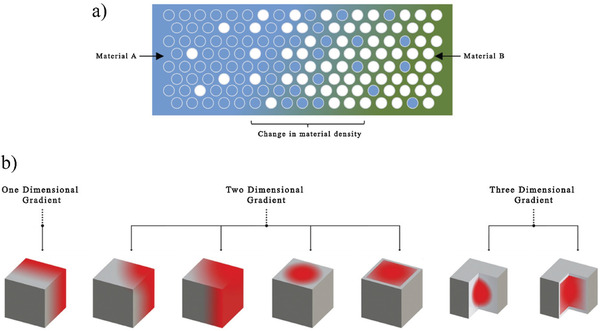
a) Schematic of combination of density and compositional gradation within a heterogeneous material, and b) types of gradients classification. Adapted with permission.^[^ ^116^ ^]^ Copyright 2018, Elsevier.

FGMs are characterized by composition variation across the part.^[^ ^48^, ^49^, ^50^ ^]^ The design of heterogeneous compositional gradients is illustrated in **Figure** **11**a and it can be categorized according to 1D, 2D, and 3D as shown in Figure 11b. Distribution of the materials can also be uniform or through special patterns.

**Figure 12:**
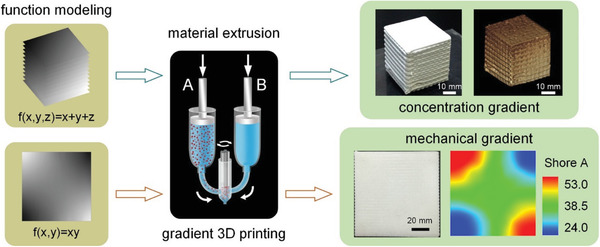
3D printing of objects with spatially non‐linearly varying properties. Reproduced with permission.^[^ ^52^ ^]^ Copyright 2018, Elsevier.

Both FDM and DIW methods have been used for extruding FGM materials. Different materials can be mixed in a static mixer to form a uniform paste. The directions of depositing each layer and gap sizes between filaments are the important printing parameters that affect the mechanical properties.^[^ ^51^ ^]^ Two identically shaped FDM models, but with different deposition densities and orientation of printing were fabricated by Li et al.^[^ ^51^ ^]^ to demonstrate the differences in stiffness along the horizontal axis. Oxman et al.^[^ ^39^ ^]^ fabricated an FGM made of soft blue silicone (Shore 00–10) mixed with a harder red silicone (Shore 00–50) to fabricate gradients in both color and durometer. Other combinations of materials were tested in their work for potential use on the platform including UV‐curable silicones and polyurethanes. Ren et al.^[^ ^52^ ^]^ fabricated polyurethane objects with various gradient patterns (see **Figure** **12**). Multiple nonlinear 1D/2D/3D color/Al_2_O_3_ concentration gradient objects were successfully manufactured. In their study, the results of the cantilever bending test and simulation showed that the material gradient can effectively relieve the stress concentration.

**Figure 13:**
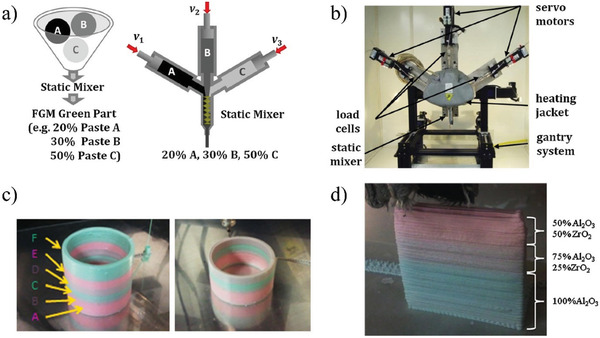
3D printing of FGMs using a triple extruder a) schematic; b) the triple‐extruder system in a temperature‐controlled enclosure: three servo motors control linear cylinders for paste extrusion and a three‐axis gantry system controls nozzle movement; c) extrusion of pink and green colored CaCO_3_ pastes. The color of the fabricated part starts at pink (c‐A) and shifts to brown (c‐B), then green (c‐C), then brown (c‐D), then pink (c‐E), and finally green (c‐F); d) a fabricated test bar that was graded from 100% Al_2_O_3_ to 50% Al_2_O_3_ + 50% ZrO_2_. Adapted with permission.^[^ ^53^ ^]^ Copyright 2012, Elsevier.

**Figure** **13** shows a freeze‐form extrusion fabrication process aimed at printing FGM 3D parts by Leu et al.^[^ ^53^ ^]^ The main concept is to mix multiple pastes according to object material composition requirements and to extrude the mixed paste to manufacture a 3D part layer by layer in an environment below the water freezing temperature. On this basis, a triple‐extruder system including the mechanical machine, electronics, and computer software have been developed by the authors. The capability of the developed system was verified by observing the transitions between green and pink colored CaCO_3_ pastes and relating them to the measured velocities of the corresponding plungers.

### 3.3 Powder Bed Fusion

Powder bed fusion (PBF) is an AM technology whereby a heat source (e.g., laser, heated printing head) is used to consolidate a material powder to form 3D parts. The heat source is applied to powder particles which gradually indexes down as each layer is finished and new powder is spread over the build area. The PBF process for polymers includes the following common printing techniques: electron beam melting (EBM), selective heat sintering (SHS), selective laser melting (SLM), and selective laser sintering (SLS).

One of the benefits of multi‐material 3D printing when compared to the standard single‐material printing is less possibility of powder cohesion which usually leads to inaccurate part dimensions and poor surface finish. Therefore, a process in which a “build” powder (e.g., a polymer) is co‐deposited with a non‐fusible “support” powder (e.g., a different polymer or ceramic) would completely avoid this issue. With multi‐material powder deposition, expensive polymer powders could be placed only where needed, and cheap, fully reusable ceramic powder would form the surroundings to provide mechanical support during the build process. It is clear that such a process could significantly reduce powder waste.

**Figure 14:**
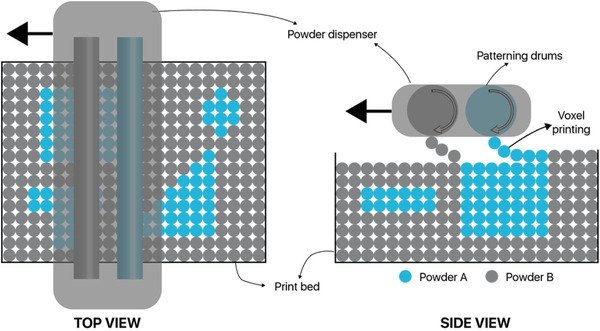
A selective powder recoating technology by Aerosint SA. Reproduced with permission.^[^ ^69^ ^]^ Copyright 2019, Aerosint SA.

Recently, Aerosint SA company (Belgium) has developed a low‐waste, multi‐material 3D printing process based on powder bed fusion technology compatible with both polymer and metal powders. Their prototype is a retrofitted industrial SLS printer in which they have integrated their patterning drums (see **Figure** **14**). The process is based on the selective deposition of voxels of powder in a layer‐by‐layer way, with sintering happening uniformly for polymers or via laser for metals that require higher temperatures. Powder bed fusion with multiple polymer powders was used in the work of some researchers.^[^ ^54^, ^55^ ^]^ Laumer et al.^[^ ^54^ ^]^ used a simultaneous laser beam melting (SLBM) technique to additively manufacture parts consisting of different polymer powders within one building process. By applying a simultaneous illumination with changeable intensity distribution over a large area, different polymeric powders deposited next to each other within a layer can be transferred simultaneously from a solid into a molten form. Nevertheless, the accurate preparation of arbitrary multi‐material powder layers to still be achieved perhaps by using advanced coating/deposition methods. Another technology to generate multi‐material powder layers can be the electrophotography also known as xerography.^[^ ^55^ ^]^ Stichel et al.^[^ ^55^ ^]^ demonstrated the application of electrophotographic polymer powder transfer for the SLS‐based preparation of multi‐material layers. An experimental setup with two chambers was designed that enabled the investigation of the electrophotographic powder transfer at typical process conditions of SLS. Their results confirmed the beneficial application of electrophotography for multi‐material powder deposition.

### 3.4 Material Jetting

**Figure 15:**
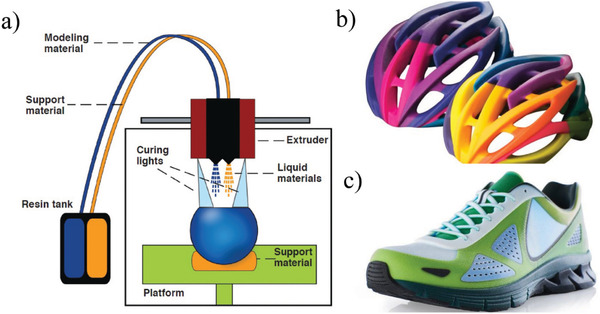
a) Schematic of material jetting process. Reproduced with permission.^[^ ^117^ ^]^ Copyright 2014, Aerosint SA, and b,c) 3D printed bicycle helmet and shoe. Adapted with permission.^[^ ^119^ ^]^ Copyright 2020, Stratasys.

Material jetting (MJ) is another AM process that can print multiple materials in the same printing job. MJ creates objects in a similar method to a 2D ink jet printer. Material is jetted onto a build platform using either a continuous or drop on demand (DOD) approach. A list of commercially available multi‐material jetting printers are listed in Table 2. PolyJet (Stratasys Ltd., USA) is probably the most common commercially available multi‐material jetting process. In this system, the nozzles are able to switch between different materials, including support material. Schematic of material jetting is shown in **Figure** **15** with the print tray and the respective print head movement (Figure 15a) and two examples of printed structures (Figure 15b,c). The hardware and software architectures for these multi‐material printers are locked‐down.

Several authors used PolyJet 3D printers for fabrication of multi‐material systems.^[^ ^56^, ^57^, ^58^, ^59^, ^60^, ^61^, ^62^ ^]^ Connex3 Objet260,^[^ ^58^, ^60^, ^61^ ^]^ Connex3 Objet350,^[^ ^59^ ^]^ Connex3 Objet500,^[^ ^59^ ^]^ and Objet1000 Plus^[^ ^57^ ^]^ were used in these studies. Some details of these printers are listed in Table 2. For instance, Keating et al.^[^ ^56^ ^]^ used a Stratasys Objet500 Connex multi‐material 3D printer to fabricate a 3D printed multi‐material microfluidic proportional valve. The developed microfluidic valves enabled the development of programmable, automated devices for controlling fluids in a precise manner. Compared to previous single‐material 3D printed valves that are stiff, the multi‐material valves developed by the authors constrain fluidic deformation spatially. This has been done through combinations of stiff and flexible materials, to enable intricate geometries in an actuated, functionally graded device. Cazón‐Martín et al.^[^ ^60^ ^]^ analyzed a novel approach that combines lattice structures and a multi‐material additive manufacturing for the design and manufacturing of soccer shin pads. The shin pads were consisting of a sandwich structure: two rigid layers (inner and outer) and a middle layer having a lattice structure that works as a shock‐absorbing geometry. A Connex3 Objet260 printer was used in their study to fabricate the specimens. The developed shin pads were dynamically tested along with two commercially available shin pads using drop weight impact tests. The results showed that two of the specimens have acceleration reductions between 42% and 68% with respect to the commercial ones, while the penetration was reduced by 13–32%.

Another commercial multi‐material jetting process is ProJet (3D Systems, USA). The main difference between PolyJet and ProJet is that PolyJet uses a water‐soluble photopolymer as the support material while ProJet uses a wax. As discussed earlier, researchers have studied the PolyJet 3D printers extensively, however, less works can be found on ProJet 3D printer in the open literature. Yang et al.^[^ ^63^ ^]^ evaluated the building performance of the ProJet 5500X multi‐material machine. The authors measured the dimensional error and surface roughness of the printed parts and analyzed them using a microscope, a 3D coordinate measuring machine, and a surface profilometer. They found that by using wax as the support material, fine features and lateral features with dimensions as small as 250 µm could all be built properly. Features with high depth and diameter ratios were also possible to be built. The authors also found that the printing accuracy of a material jetting system mainly affected by the accuracy of the printer machinery (such as droplet size and print heat positioning), material property such as shrinkage, and the size and structure of product.

Electrohydrodynamic jet (E‐jet) has also been adapted for multi‐material printing using a multi‐nozzle head. E‐jet is a high resolution material jetting printing technology where the printed liquids are driven by an electric field. E‐jet printed droplet ranges from nano‐ to micro‐scales. During the past decade, there has been various applications for E‐jet printing, primarily for biosensing and printed electronics applications. Pan et al.^[^ ^64^ ^]^ proposed a multi‐level voltage approach to perform the addressable E‐jet printing utilizing multiple nozzles in parallel with high consistency. The multi‐level voltage approach controls the electric field on each of the nozzles. A good dimensional and position consistency was observed in the printed objects. The authors showed that multi‐level voltage approach is an efficient way to perform the addressable E‐jet 3D printing with several parallel nozzles with high consistency.

**Figure 16:**
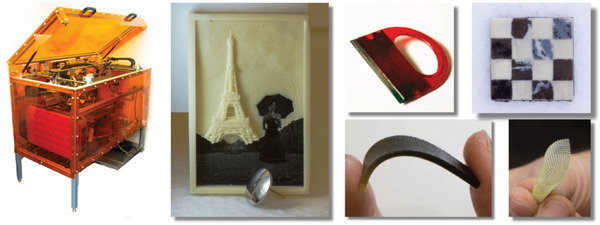
(left) Multifab multi‐material 3D printer and, (right) a set of fabricated materials and objects. Reproduced with permission.^[^ ^17^ ^]^ Copyright 2015, ASSOCIATION FOR COMPUTING MACHINERY.

Custom‐made material jetting printers can also be found in the literature.^[^ ^65^, ^66^ ^]^ MultiFab (see **Figure** **16**) is a machine vision assisted platform for multi‐material 3D printer developed by Sitthi‐Amorn et al.^[^ ^17^ ^]^. MultiFab can print simultaneously up to ten different materials. The platform achieves a resolution of at least 40 µm by utilizing piezoelectric inkjet printheads adapted for 3D printing. Unlike previously discussed commercial printers, the hardware and software architectures of MultiFab are extensible and reconfigurable. Moreover, none of the commercial 3D printers uses machine vision system for calibration, 3D scanning, closed feedback loop, and alignment with auxiliary objects.

### 3.5 Sheet Lamination

Laminated object manufacturing technique (LOM) includes layers of adhesive‐coated paper, plastic, or metal laminates that are successively glued together and cut to shape with a knife or laser cutter. Sheet lamination process categories based on the mechanism employed to achieve bonding between layers are gluing or adhesive bonding, thermal bonding, clamping, and ultrasonic welding. Sheet lamination approaches exhibit the speed benefits of a layer‐wise process while still utilizing a point‐wise energy source. In the multi‐material LOM, the material feed comes from dissimilar materials.^[^ ^3^ ^]^ Limited studies have focused on the application of multi‐material LOM. For instance, Mohammadzadeh et al.^[^ ^67^ ^]^ combined xurography^[^ ^68^ ^]^ with LOM to create multi‐material microfluidic devices. In their process, 2D layers were placed upon each other to fabricate a 3D object.

## 4 Multi‐Material Additive Manufacturing of Metals and Ceramics

### 4.1 Powder Bed Fusion

As discussed in Section 3.3, powder bed fusion machines use thermal energy such as laser for melting the powder into the designed shape. Commonly used printing techniques in powder bed fusion are SLS, SLM, EBM, and direct metal laser sintering (DMLS). One of the major disadvantages of current powder bed fusion methods is that they are inherently mono‐material. The current focus of AM is to simplify and streamline manufacturing by enabling the production of geometrically complex, functional parts that can effectively replace the entire assemblies made from many simple components. Such assemblies are often made of a variety of materials. Hence, a future direction for AM of metals should be to produce parts made of multiple materials. Currently, the patented spatially selective, multiple‐powder deposition system of Aerosint SA (Belgium) seems to be the only available multi‐material 3D printing system based on powder bed fusion technology adaptable to metal, ceramic, and polymer powders (see Figure 14).^[^ ^69^ ^]^

##### FGMs in Powder Bed Fusion of Metals and Ceramics

The powder bed fusion methods such as SLS can be also used to produce multi‐material FGM parts. Based on SLM technology, Mumtaz et al.^[^ ^70^ ^]^ fabricated an FGM component blending Waspaloy and Zirconia materials using a high powered laser. The graded specimens initially consisted of 100% Waspaloy with subsequent layers containing increased volume compositions of Zirconia (0–10%). It was found that specimens contained an average porosity of 0.34% and a gradual change between layers without any major interface defects.

### 4.2 Directed Energy Deposition

Directed energy deposition (DED) is an AM process in which, the focused thermal energy is used to bond materials by melting as they are being deposited. Powder feed and wire feed systems are two major subcategories of DED. Other popular terms for DED include laser engineered net shaping (LENS), directed light fabrication (DLF), direct metal deposition (DML), laser metal deposition (LMD), laser deposition welding (LDW) and 3D laser cladding, Wire+Arc additive manufacturing (WAAM). The build volumes of these systems are generally larger than powder bed fusion. Various metallic alloys are available and it is possible to gradually and continuously change from a material to another one while manufacturing. This particularity makes possible the manufacturing of multi‐material parts.

The multi‐material DED can encompass several different technologies that are identified by the way the material is being fused, each suited for different and specific purposes. The techniques based on powder bed fusion technology can only create discrete material gradient. The DED, on the other hand, is capable of fabricating multi‐material with continuous gradient within and across the layers. However, parts made by DED require multiple steps of post‐processing to acquire desired shape and dimensional accuracy. Li et al.^[^ ^71^ ^]^ employed the SLM technique for AM of 12 wt% nano TiN‐modified CoCrFeNiMn. The TiN nanoparticles led to a uniform distribution in the FCC (face‐centered cubic) matrix.

#### 4.2.1 Powder Feed Systems

**Figure 17:**
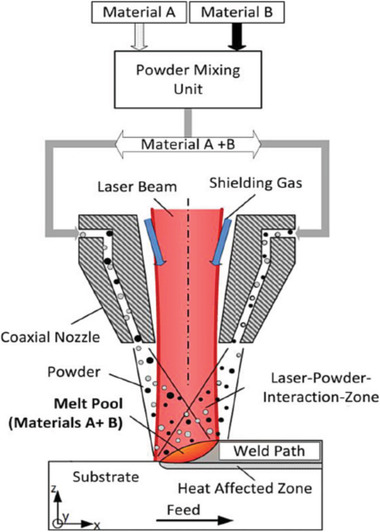
Powder fed multi‐material LMD processing: several different powder materials (e.g., Material A and B) can be mixed in situ by an integrated powder‐mixing chamber in the nozzle tip. Reproduced with permission.^[^ ^75^ ^]^ Copyright 2019, AIP Publishing.

The powder feed technologies (such as LENS) use thermal energy (e.g., laser) to print parts layer by layer from metals, alloys, ceramics, or composites in powder form. The LENS technology has been used to fabricate FGM objects such as the composite of stainless steel 316L and Stellite Grade 12 Co‐Cr alloy.^[^ ^72^ ^]^ Both continuous and sharp/discrete compositional gradient parts could be fabricated in periodic multilayered structures, and the transition zone thickness was controllable by process variables. Muller et al.^[^ ^73^ ^]^ modeled a powder flow rate by a first order transfer function with the capability of material composition in each layer to be adjusted by varying the powder flow rate of different primary materials. AM of an Inconel 718‐Copper alloy bimetallic structure was studied by Onuike et al.^[^ ^74^ ^]^ using LENS. The bimetallic structure was fabricated with the goal of improving the thermal and mechanical properties compared with the Inconel 718 alloy. The average thermal diffusivity of the bimetallic structure was measured at 11.33 mm^2^ s^−1^ for the temperature range of 50–300 °C; a 250% increase in diffusivity was observed when compared to the pure Inconel 718 alloy at 3.20 mm^2^ s^−1^. Conductivity of the bimetallic structures increased by almost 300% compared to Inconel 718 as well. Brueckner et al.^[^ ^75^ ^]^ used a similar technique to fabricate linearly graded material combination SS AISI 316L and INC718 (**Figure** **17**). Their studies showed that the linearly graded transitions for combining SS AISI 316L and INC718 were beneficial using LMD.

#### 4.2.2 High‐Entropy Alloys

High‐entropy alloys (HEAs) are formed by mixing equal or relatively large proportions of five or more elements.^[^ ^76^, ^77^, ^78^ ^]^ The HEAs are popular for their superior properties such as better strength‐to‐weight ratios, with a higher degree of fracture resistance, tensile strength, as well as corrosion and oxidation resistance than conventional alloys.^[^ ^79^ ^]^ Hence, HEAs are expected to be high‐performance novel structural materials, substituting for conventional alloys such as Ni‐based superalloys and stainless steels.^[^ ^79^ ^]^

**Figure 18:**
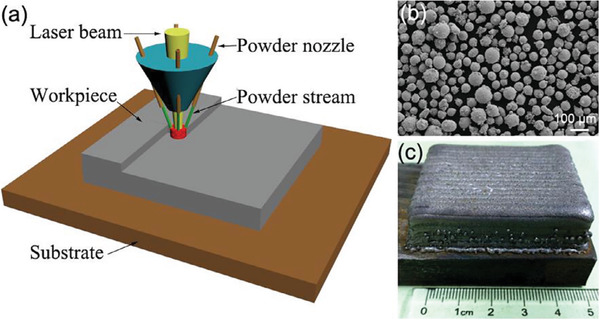
a) A schematic of coextrusion of powders for printing of HEA; b) microstructure of the printed HEA; c) as‐printed CoCrFeMnNi HEA sample. Reproduced with permission.^[^ ^76^ ^]^ Copyright 2019, Elsevier.

Some research works have been performed on AM of parts made of HEAs.^[^ ^71^, ^76^, ^79^, ^80^, ^81^ ^]^ For instance, Gao and Lu^[^ ^76^ ^]^ used a coaxial powder feeding laser 3D printing system (see **Figure** **18**) to print CoCrFeMnNi alloys. The authors investigated the microstructure (Figure 18b) and mechanical properties of fabricated HEA. An equiaxed‐to‐columnar transition structure was observed in the melt pool of the printed sample. The printed HEA exhibited an outstanding combination of high strength and excellent ductility. The ultimate tensile stress of the printed CoCrFeMnNi HEA was stronger than that of the as‐cast alloy while its ultimate tensile elongation was comparable.

#### 4.2.3 Wire Feed Systems

**Figure 19:**
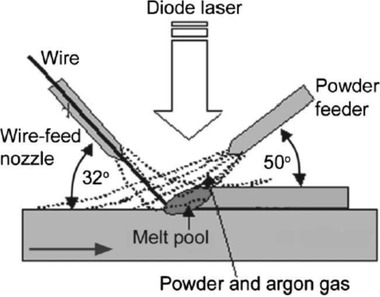
Simultaneous wire‐ and powder‐feed direct metal deposition. Reproduced with permission.^[^ ^82^ ^]^ Copyright 2006, AIP Publishing.

In multi‐material wire feed direct deposition, wires of desired materials are fed and then melted using an energy source (laser or an electron beam). The energy source solidifies the wires on the bed along a preferred path. The part is then built in a layer‐by‐layer fashion until a complete component is made. Syed et al.^[^ ^82^ ^]^ investigated the process characteristics of simultaneous wire‐ and powder‐feed direct metal depositions for possible higher build rate and higher material usage efficiency while maintaining the geometry accuracy (see **Figure** **19**). Their study compared the process characteristics, advantages and disadvantages of wire‐ and powder‐feed DED and showed that by adding powder and wire, the deposition rate can be increased.

##### FGMs in DED of Metals and Ceramics

**Figure 20:**
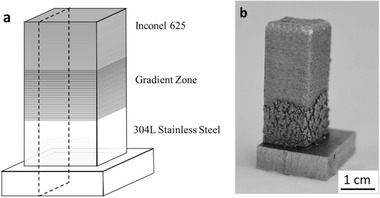
a) Schematic of gradient alloy specimen, b) Photograph of specimen after fabrication by laser‐based powder feed DED. Reproduced with permission.^[^ ^83^ ^]^ Copyright 2016, Elsevier.

Fabrication of metal and ceramic FGMs in DED is usually performed by using multiple chambers with different powder materials to be deposited on different layers in order to make the desired FGM component. Caroll et al.^[^ ^83^ ^]^ fabricated an FGM part by powder‐feed DED with an FGM structure from SS304L to the nickel‐based alloy IN625 (**Figure** **20**). Microparticles of a secondary phase responsible for development of cracks in fabrication and microhardness were observed near the SS304L end of the gradient zone (≈82 wt% SS304L).

### 4.3 Sheet Lamination

Ultrasonic AM (UAM) is a solid‐state metal seam welding method that utilizes sound to bond layers of metal. The technique creates strong bonds with high density and works with different metals. UAM was used by several authors to produce metal FGMs.^[^ ^84^, ^85^, ^86^ ^]^ For instance, Kumar^[^ ^84^ ^]^ studied the joining of different metallic foils using stainless steel, Al, and Cu foils. The foils were joined by ultrasonic welding using a machine that mechanically vibrates the welding head at 20 kHz. In the work of Bisadi et al.,^[^ ^87^ ^]^ by UAM, the authors lap joined dissimilar sheets of 5083 Al alloy and commercially pure copper method. It was shown that joint defects appear at very low or high welding temperatures.

## 5 Multi‐Material Additive Manufacturing of Biomaterials

In a broader prospective, use of AM for printing tissues and organs made of biomaterials can be classified as a) biomaterials without cell (acellular biomaterials) such as scaffolds made of natural or synthetic polymers and b) biomaterials with cell (cellular bio‐inks). Once the scaffold is printed, the cells are deposited using a 3D printing technology. Bio‐ink containing live cells controls positioning and the amount of cells.

Natural polymers (e.g., alginate‐gelatin, collagen, chitosan, cellulose) are beneficial for manufacturing of scaffolds, but synthetic polymers (e.g., polycaprolactone [PCL], ABS, PLA, PA, polydimethylsiloxane [PDMS], polyether ether ketone [PEEK]) are sometimes preferred for their high mechanical strength, controlled degradation rate, and processability. Ceramic polymers (e.g., hydroxyapatite) can be used for fabricating scaffolds for bone regeneration due to their desired mechanical properties and biocompatibility. Bioceramic scaffolds have bioactive component to support the growth of bones.

**Figure 21:**
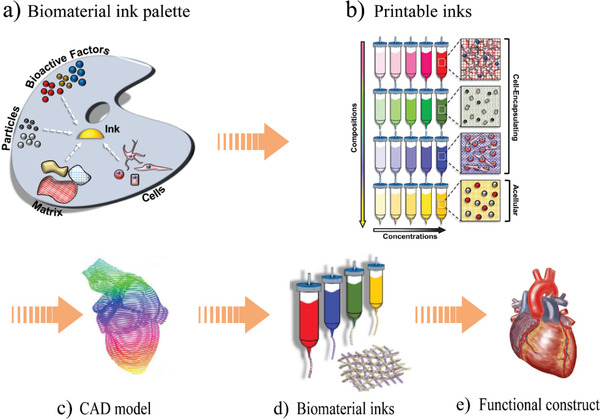
Schematic of multi‐material bioplotting of biomaterials: a) a biomaterial ink palette for fabrication of tissues and organs, b) printable inks with different compositions, c) computer model of an organ, d) biomaterial inks, and e) an example of a functional construct. Reproduced with permission.^[^ ^97^ ^]^ Copyright 2016, IOP Publishing, Ltd.

The main technology used for deposition and patterning of multiple biomaterials is extrusion, also known as bioplotting.^[^ ^88^, ^89^, ^90^, ^91^, ^92^, ^93^, ^94^, ^95^ ^]^ Bioplotting is based on extruding a material with specific viscosity from a syringe to fabricate 3D shape of biomaterials, as shown in Figures 6 and **21**. Bioplotting allows for the production of a wide variety of practical biomedical tissues with different shapes and material compositions.^[^ ^96^ ^]^ The extrusion system based on syringe achieves relatively low resolution. However, the key advantage of this technique is material flexibility. Biomaterials in the form of pastes, solutions, and hydrogels can all be fed into 3D bioplotters. A temporary, sacrificial material may be needed to support the printed structure since viscous raw materials have low stiffness that may result in the collapse of complex structures. A list of commercially available bioplotters is provided in Table 3.

Multi‐material 3D printed scaffolds, tissues, and organs require different bio‐inks, all of which must demonstrate cell‐compatibility and printability. For instance, the printing and post‐processing of the acellular ink should be cell‐compatible for multi‐material printing of cellular and acellular inks. Therefore, the use of organic solvents or extreme temperatures is not recommended as it would compromise cell viability within the printed structure.^[^ ^97^ ^]^

**Figure 22:**
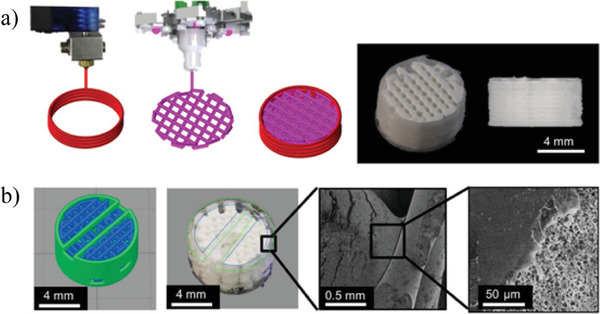
a) Combinatorial printing process with layer by layer deposition of the thermoplastic polyester outer shells and high internal phase emulsions (HIPE) emulsion ink inner material; b) integration between the emulsion ink and thermoplastic (PCL) shell. Adapted with permission.^[^ ^95^ ^]^ Copyright 2017, Elsevier.

Bakarich et al.^[^ ^92^ ^]^ developed a material extrusion‐based gradient printing system, and its function was demonstrated by 3D printing a range of tough hydrogel composites. A spectrum of soft and wet to hard and dry particulate‐reinforced composites were prepared by changing the ratios of a soft alginate/polyacrylamide‐based hydrogel to a hard UV‐curable ink in the materials. The printed materials were mechanically characterized in tension and were modeled by composite laminate theory. Sears et al.^[^ ^95^ ^]^ reported the development of a biodegradable, fumarate‐based emulsion ink for bioprinting robust bone grafts with designed, hierarchical porosity. A combinatory approach that utilized thermoplastic polyester printing to reinforce the emulsion ink prints was then developed by the authors to enhance the compressive properties and illustrate the potential of this technique to improve scaffold biomimicry. In the authors’ work, the addition of either a PCL or PLA shell resulted in a significant increase in compressive modulus and yield strength with the PLA shell resulting in constructs with compressive properties in the range of trabecular bone (see **Figure** **22**). A multichannel open source hardware and software 3D bioplotter was designed by Lee et al.^[^ ^93^ ^]^. Hybrid scaffolds with synthetic polymeric materials and cell laden hydrogels were printed and the authors verified the performance of the 3D bioplotter.

Multi‐material 3D printing of inkjet‐based systems were also used for 3D printing of biopolymers. Poellmann et al.^[^ ^98^ ^]^ 3D printed micropatterned, multi‐material hydrogrels using E‐jet direct ink writing. The authors fabricated polyacrylamide features in microscale integrated in another hydrogel of a different composition. Once photopolymerization was done, the droplets were backfilled with a second polyacrylamide mixture, the second mixture was polymerized and the sample was peeled off the substrate. Fluorescent and confocal microscopies verified multi‐material patterning, while scanning probe microscopy revealed a patterned topography with printed spots forming shallow wells.

## 6 Multi‐Material 4D Printing

Multi‐material 4D printing uses AM technologies to fabricate stimulus‐responsive parts that can actively change their properties when subject to appropriate stimuli. The use of 4D printing is expected to become more popular with applications across biomedical, aerospace, and electronic industries.

Geometrical transformation after 3D printing is the main feature of multi‐material 4D printing. Shape changes (or shape memory effect) in 3D printed parts can be induced by different external stimuli, to cause shrinkage, expansion, or folding of the printed parts as the fourth dimension. Shape memory polymers (SMPs) and shape memory alloys (SMAs) are two different kind of materials that are utilized for 4D printing. SMPs may be preferred over SMAs due to their wide range of glass transition temperatures, *T*_g_, from −70 to 100 °C, permitting their elastic properties to be tailored.^[^ ^99^ ^]^ Some common problems with SMAs are their complex manufacturing, higher costs, toxicity, and limited recovery. For instance, SMPs can obtain a shape recovery property up to four times of plastic strain, whereas SMAs are around 7–8%. The challenge for multi‐material 4D printing is to utilize materials that are strong and malleable in the presence of stimuli. Ideally, when material is induced by different stimuli should also exhibit different behavior.^[^ ^100^ ^]^

**Figure 23:**
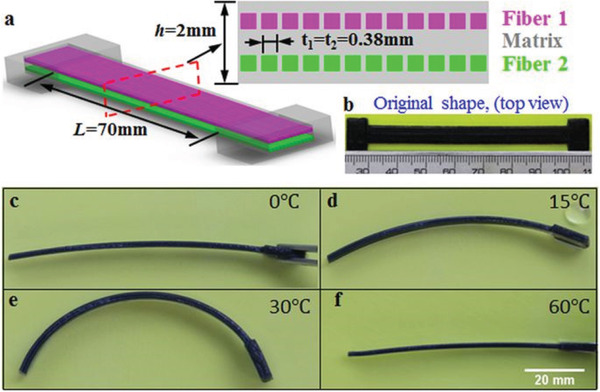
Multi‐shape memory effects of a printed active composite strip: a) the design and dimensions of the sample. The enlarged drawing is the cross section of the structure. b) The original printed sample. The length scale in the bottom is in mm. c–f) Shape change of the sample at different temperatures. Reproduced with permission.^[^ ^101^ ^]^ Copyright 2016, Springer Nature.

**Figure 24:**
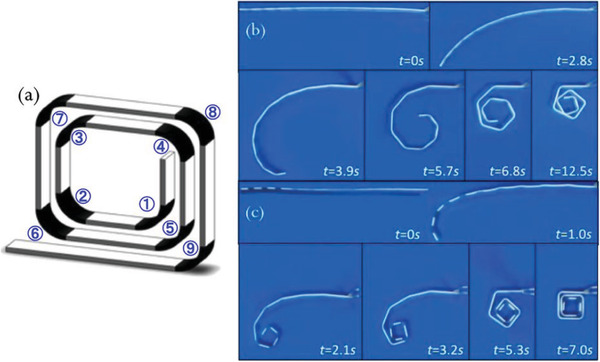
a–c) The schematic of sequential self‐folding strand. Series of photographs showing the shape recovery process of the helical SMP component. Reproduced with permission.^[^ ^102^ ^]^ Copyright 2015, Springer Nature.

From stimulus point of view, multi‐material 4D printing can be classified by their stimuli like temperature, humidity, or solvents, as well as pH or light. Wu et al.^[^ ^101^ ^]^ used different thermal response SMP fibers to print composite materials having different shape changes with rising temperature. The authors used two fibers with different (*T*_g_ of ≈57 and ≈38 °C). After a simple single‐step thermomechanical programming process, the fiber families could be sequentially activated to bend when the temperature was increased. **Figure** **23** shows the active motion of printed objects consisting of SMP fibers that have different *T*_g_. Mao et al.^[^ ^102^ ^]^ utilized multiple thermal response SMPs with different *T*_g_ to fabricate more complex motion of printed parts using Objet Connex 260. A composite strand was printed with hinges comprised of nine SMPs for shape recovery process sequentially. **Figure** **24** indicates the folding of the 1D strand with the hinge section after immersing in hot water (≈90 °C). Bodaghi et al.^[^ ^103^ ^]^ designed and fabricated a self‐expanding/shrinking mechanism by fabricating two types of SMPs with low and high *T*_g_ in fiber forms into a flexible matrix. SMP fibers were eccentrically located in the beam‐type actuator unit and their arrangement was changed along the beam length. Self‐expansion and shrinkage characteristics were verified both experimentally and numerically. The authors fabricated planar and tubular shapes composed of periodically arranged actuating units. The actuating units were made from TangoBlackPlus and VeroWhitePlus available in the Objet 500 printer material library. In another study, Bodaghi et al.^[^ ^104^ ^]^ explored 4D printing of triple SMPs with self‐bending feature. The concept was on the basis of arranging hot–cold programming with FDM printing technology to engineer triple SMPs. Their experiments revealed that the printed SMPs have elasto‐plastic response at low temperatures while they behave hyper‐elastically at high temperatures in the large deformation regime.

**Figure 25:**
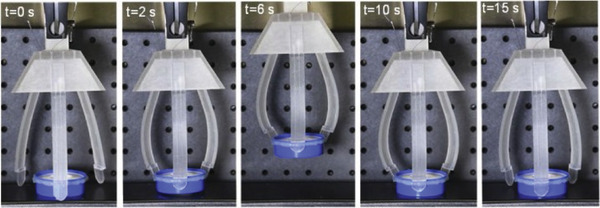
Snapshots of grabbing an object using a multi‐material printed bending soft actuator by embedding thin SMA wires eccentrically into a polymeric soft matrix (FLX9940). Reproduced with permission.^[^ ^107^ ^]^ Copyright 2019, Elsevier.

Using a high resolution PµSL and incorporating a family of photo‐curable copolymer networks, Ge et al.^[^ ^105^ ^]^ printed high‐resolution multi‐material thermal response SMPs. They fabricated a multi‐material gripper and the microscale resolution achieved using the PµSL method. Owing to photo‐curable thermoset with different crosslinkers, the authors could print devices with a variety of *T_g_*. The same research group in Singapore, has extended the concept of multi‐material 4D printing to active hinges^[^ ^106^ ^]^ and SMA wires.^[^ ^107^ ^]^ For instance, Akbari et al.^[^ ^107^ ^]^ fabricated soft actuators by embedding SMA wires into various soft matrices manufactured by multi‐material 3D printing (see **Figure** **25**). In order to achieve a wide range of deformations, ten different printing materials were characterized and used in their actuators design. In addition, the authors developed a finite element model to simulate complex deformations of the printed actuators and facilitate the design process.

**Figure 26:**
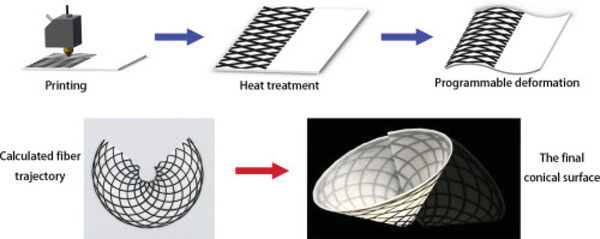
4D printing method by embedding continuous fibers in matrix, realizing deformation of complex surfaces. Reproduced with permission.^[^ ^108^ ^]^ Copyright 2018, Elsevier.

Another challenge in the field of 4D printing is the controllable morphing. In a study conducted by Wang et al.^[^ ^108^ ^]^ 4D printing of continuous carbon fiber‐reinforced composites was introduced. The composite fabricated by this method could realize programmable deformation with a high deformation accuracy (see **Figure** **26**). The deflection of the printed composite was initiated by the difference of coefficients of thermal expansion (CTEs) between continuous carbon fibers and flexible Polyamide66 matrix.

**Figure 27:**
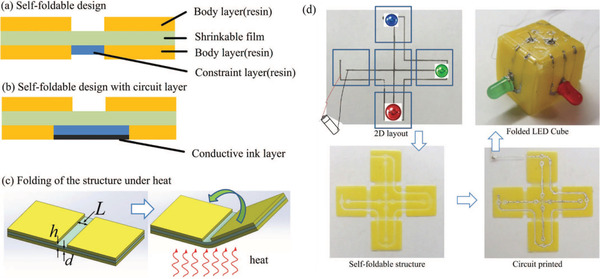
**S**elf‐foldable 4D printing using DIW a) self‐foldable design and b) folding of structure under heat, c) LED cube case produced by self‐folding mechanism after printing. Reproduced with permission.^[^ ^109^ ^]^ Copyright 2016, IEEE.

Moreover, 4D printing technique can provide the opportunity of printing 3D electronic circuits. Deng et al.^[^ ^109^ ^]^ designed a mechanism (**Figure** **27**a) to obtain self‐folding 3D circuits (Figure 27b) using a polystyrene film sensitive to thermal stimuli based on DIW method. As depicted in Figure 27a, resin was used for one side of the film as a constraint layer, and the other side of the film was left empty. As a result, by rising the temperature, the empty side was folded on the hinge.

Simulation of multi‐material 4D printed objects can also be found in the literature.^[^ ^104^, ^110^, ^111^ ^]^ For instance, to enhance design capabilities of dual/triple SMP by 4D printing, a phenomenological constitutive model was developed by Bodaghi et al.^[^ ^104^ ^]^. The authors incorporated crucial elements including SMP phase transformation, hyper‐elasticity, elasto‐plasticity, and hot‐cold programming in the framework of large deformation regime in their models. Their developed model was then coupled with the finite element formulation to simulate 4D printed dual/triple SMP structures through an elastic‐predictor plastic‐corrector return map algorithm. The experimental and numerical results in their study demonstrated the potential applications of dual/triple SMPs in mechanical and bio‐medical engineering devices like self‐bending gripers/stents and self‐shrinking/tightening staples. Sossou et al.^[^ ^110^ ^]^ proposed a modeling framework for simulating smart and conventional materials behaviors on a voxel basis. This allowed for arranging materials in any distribution and rapidly evaluating the distribution behavior. Homogeneous and heterogeneous objects made of conventional and smart materials were modeled and simulated in their work. A printed smart valve and a theoretical actuator were used as test cases in the authors’ work.

## 7 Discussion, Evaluation, and Future Directions

Many different 3D and 4D printing processes for fabrication of multi‐material polymer, metal, ceramic, and biomaterials have been reviewed in this manuscript. It was noticed that the vat photopolymerization process is not generally a candidate for multi‐material 3D printing. It prints parts from a vat of UV curable resins, and the use of multiple materials in vat photopolymerization provides challenges with contamination management between material systems. However, due to its advantages such as high‐quality surface finish, dimensional accuracy, and a variety of material options that includes transparent materials, vat photopolymerization has been adapted to support multi‐material printing. The material extrusion technique can be easily extended to multi‐material 3D printing through the use of multiple nozzles. PBF methods also suffer from inherent mono‐material processability. A future direction for AM of metals should be to produce parts made of multiple materials. Currently, the patented spatially selective, multiple‐powder deposition system of Aerosint SA (Belgium) seems to be the only available multi‐material 3D printing system based on powder bed fusion technology adaptable to metal, ceramic, and polymer powders. Material jetting is a common method for multi‐material printing. PolyJet (Stratasys Ltd., USA) is probably the most common commercially available multi‐material jetting process. The hardware and software architectures for these multi‐material 3D printers are often proprietary and inextensible. Due to the complicated technology, less custom‐made 3D printer based on material jetting is available (e.g., Multifab ^[^ ^65^ ^]^). To the best of authors’ knowledge, no work on multi‐material 3D using binder jetting was found in the literature.^[^ ^112^ ^]^ DED methods seem to be good candidate for multi‐material printing. The build volumes of these systems are generally larger than powder bed fusion. Various metallic alloys are available and it is possible to gradually and continuously change from a material to another one while manufacturing. Powder feed and wire feed systems are two major subcategories of DED, however, no work on multi‐material 3D and 4D printing using wire feed system was found in the literature. In multi‐material LOM, the material supply either comes from two different materials or comes from blended multiple materials. However, the studies focused on the application of multi‐material LOM are very limited as discussed in the text.

Advanced 3D printed composite materials and 4D printed responsive materials were described with their specificities and their ontologies. Moreover, various functions of these products in different industries including medical devices, electronics, biomedical implementations, sporting goods, and robotics have been summarized. It was seen that part performance could be enhanced by the utilization of multiple material systems and complex geometries. The use of composites can also target functional regions within a part; applying the most appropriate materials in the most appropriate areas. Topological optimization is employed to propose new complex geometries which are easier to fabricate by AM with weight gain while keeping a relatively high mechanical behavior. The predictive computational models to simulate the geometry of 3D‐4D printing filaments is currently being studied to improve the properties of multiple material systems.

Although considerable advancements were achieved via 3D and 4D multi‐material printing, the potential has not been fully explored yet.

### 7.1 Mechanical Properties

The mechanical performance of multi‐material additively manufactured parts is usually better in comparison with those printed by single‐material printing. Formation of voids between subsequent layers of printed parts can affect their mechanical performance due to a decrease in interfacial bonding between printed layers. Different mechanical behavior under vertical tension or compression compared to that of the horizontal direction is another common challenge of multi‐material AM. Robust 3D printing processes such as micro‐additive layering are important to provide stability between layers and improve surface finish to the resolutions that meet their specific applications.

### 7.2 Production Efficiency

Efficiency in production of parts may be another potential research direction in multi‐material AM systems. A balance between the production efficiency (e.g., production rate) and part quality is needed all the time. One may use a higher energy power or faster scanning speed to raise the production rate, but the part quality may be influenced. To overcome this problem, one solution is to optimize the printing parameters. Another issue that affect the production efficiency is the complex post processing methods. Effective methods for post‐processing, including support material removal and processes related to heat treatment, need to be improved.

### 7.3 Micro/Nano Multi‐Material AM

While the majority of commercial multi‐material 3D printers create parts in macroscale, variety of sizes may be needed. Recently, micro/nano multi‐material AM processes have attracted a significant attention because of their influence on many applications such as MEMS/NEMS and nanomanufacturing.^[^ ^113^ ^]^ As discussed earlier, the electrohydrodynamic printing technique seems to be a promising printing method for 3D printing of parts from micro to nanoscale of different materials.^[^ ^114^ ^]^

### 7.4 Multi‐Material Metamaterials and Lattice Structures

Some research groups are concentrated on AM processes with multi‐material manufacturing capabilities and new internal structures based on high performance computers and optimization tools. Metamaterials and advanced lattice structures have applications in flexible materials that seem to have potentials in variety of disciplines such aerospace, civil, textile, and tissue engineering applications. The utilization of micro/nano multi‐material AM with metamaterials and lattice structures can lead to innovative functional parts.

### 7.5 AM of Continuous Fiber‐Reinforced Composites

As indicated by several researchers, the mechanical performance of continuous fiber‐reinforced composites is expected to be more significant than short fibers. Existing challenges are mainly related to processing of the materials, the bonding between fibers and the matrix, and interlaminar properties of the 3D printed continuous fiber‐reinforced composites. Choice of the appropriate 3D printing technology and finding proper binder are essential to achieve a better mechanical performance.

### 7.6 4D Multi‐Material Printing

This approach is a relatively novel and fascinating research area and has great capability for extension. As 4D multi‐material printing rooted in the 3D one, some similar challenges such as limited choice of materials, printing resolution, slow, mechanical performance, and potential to obtain dimensional accuracy. New developments could result in smart structures for actuation or motion following a predetermined program. FGMs can also tailor the microstructure properties of a 4D printed part to create more complex geometrical transformations by strategically controlling the density and directionality of stimuli‐responsive materials. It can also improve the inter‐material bonding of heterogeneous smart compositions, and even disregard the material properties of being active or non‐active. The multi‐material 4D printing development is the decisive point to accelerate the growth of the smart material area.

In summary, there are many significant achievements of multi‐material AM technology, while, some challenges still remain, including the production efficiency, mechanical properties, and applications that are mentioned above. Multidisciplinary research and development will be crucial to overcome those challenges and fully realize the potential of multi‐material AM in different applications.

## Conflict of Interest

The authors declare no conflict of interest.
